# CRISPR-Cas9 gene editing for hereditary angioedema: current treatments and emerging therapies

**DOI:** 10.1097/MS9.0000000000004270

**Published:** 2025-11-07

**Authors:** Laiba Jalal, Muskan Asim Taimuri, Anusha Sumbal, Areeba Ikram, Tehreem Ali, Ayesha Khan, Safa Alam, Umulkhairah Onyioiza Arama, Hermann Yokolo

**Affiliations:** a Department of Internal Medicine, Dow University of Health Sciences, Karachi, Pakistan; b Department of Internal Medicine, Karachi Medical and Dental College, Karachi, Pakistan; c Department of Internal Medicine, College of Medical Sciences, Ahmadu Bello University, Kaduna State, Nigeria; d Department of Research, Medical Research Circle (MedReC), Goma, DR Congo

**Keywords:** CRISPR, hereditary angioedema, CRISPR-Cas9 gene therapy, emerging therapies

## Abstract

Hereditary angioedema (HAE) is a rare autosomal dominant disorder marked by episodic, non-urticarial swelling due to C1 esterase inhibitor (C1-INH) deficiency or dysfunction, leading to excessive bradykinin-mediated vascular permeability. While current treatments focus on symptomatic control using C1-INH replacement or kallikrein inhibitors, they require frequent administration and do not address the underlying genetic defect. CRISPR-Cas9 gene-editing technologies, particularly the investigational therapy NTLA-2002, offer a transformative approach by targeting the *KLKB1* gene to durably reduce plasma kallikrein levels. This narrative review summarizes the pathophysiology and conventional treatments of HAE, and highlights emerging clinical evidence supporting the safety and efficacy of NTLA-2002. Preliminary trials demonstrate substantial reductions in HAE attack frequency and plasma kallikrein, with minimal adverse events. However, concerns about long-term safety, off-target effects, ethical implications, and accessibility remain. CRISPR-based therapeutics such as NTLA-2002 represent a paradigm shift in the management of HAE and underscore the broader potential of *in vivo* gene editing for genetic disorders.

## Introduction

Hereditary angioedema (HAE) is a rare, autosomal dominant disorder characterized by recurring, painful, and potentially life-threatening episodes of non-urticarial swelling, commonly involving the skin, gastrointestinal tract, upper airway, face, and throat^[1,2]^. Symptoms typically begin in childhood with recurrent attacks lasting hours to days^[1]^. Laryngeal edema poses a life-threatening risk of airway obstruction and asphyxiation^[1]^. HAE results from a deficiency or functional impairment of the C1 esterase inhibitor (C1-INH), which exerts inhibitory effects on kallikrein and other targets^[3]^. Consequently, C1-INH deficiency leads to increased kallikrein and bradykinin, vasodilation, increased permeability, and angioedema^[3]^. With an estimated prevalence of approximately 1 in 50 000, the disease imposes a significant burden through impaired quality of life, delayed diagnosis, and lifelong dependence on costly prophylactic or on-demand therapies, creating an urgent need for curative approaches^[4,5]^. Available treatment options for HAE involve intravenous or subcutaneous administration of C1-INH or kallikrein inhibitors. These medications are short-lived in the body, requiring long-term dosing, and focus solely on symptom management and prophylaxis, highlighting an ongoing need for better therapeutic options^[3]^. The development of NTLA-2002/CRISPR-Cas9 gene therapy is a revolutionary advancement since its mechanism of action causes decreased production of plasma pre-kallikrein, ultimately leading to a reduced total plasma kallikrein level^[1]^. NTLA-2002 reduces plasma kallikrein levels by CRISPR–Cas9–mediated editing of the *KLKB1* gene, delivered via lipid nanoparticles for targeted hepatocyte uptake and *in vivo* genome modification^[1,3]^. In a clinical trial conducted by Hilary *et al*^[1]^, dose-dependent plasma kallikrein reductions were observed (−67% at 25 mg, −84% at 50 mg, and −95% at 75 mg) from baseline through the latest assessment, while the mean percentage change in the number of monthly attacks was −95%^[1,3]^. The available literature lacks comprehensive analyses that compare conventional HAE treatments with advancing CRISPR-Cas9-based therapies like NTLA-2002, despite initial promising results. Thus, in this review, we discuss the current treatment options for HAE, while highlighting the advancing therapies, particularly the novel *in vivo* gene editing therapy NTLA-2002, which is based on the CRISPR-associated protein 9 (Cas9). This article complies with the TITAN 2025 guidelines – repressing the reporting and use of artificial intelligence (AI)^[6]^.

**HIGHLIGHTS**

- NTLA-2002 offers one-time CRISPR-Cas9 treatment for hereditary angioedema (HAE).
- Clinical trials show up to 95% reduction in HAE attacks with a single NTLA-2002 dose.
- Gene editing targets the root cause of HAE – no more lifelong symptom management.
- Early results show durable kallikrein suppression with minimal adverse effects.
- NTLA-2002 signals a breakthrough in *in vivo* gene editing for rare genetic diseases.

## Methodology

This narrative review was conducted in accordance with the PRISMA guidelines. A comprehensive literature search was conducted using electronic databases, including PubMed, Google Scholar, and Scopus. Relevant studies, including clinical trials, reviews, and original research articles, were identified using keywords such as “CRISPR,” “hereditary angioedema,” and “CRISPR-Cas9 gene therapy.” Studies published up to 2025 were included. Only studies published in English and available in peer-reviewed journals were included. Research focusing on CRISPR-Cas9 gene therapy for HAE, providing data on efficacy, safety, or mechanisms of CRISPR gene editing, was also considered for inclusion. Studies in languages other than English or those not available in full-text format were excluded.

## Pathophysiology of HAE

HAE is a rare autosomal dominant condition most commonly caused by mutations in the SERPING1 gene, which codes for C1 esterase inhibitor (C1-INH). C1 INH is a key modulator of multiple cascades, including the complement, contact, coagulation, and fibrinolytic systems. Deficiency or dysfunction of C1-INH leads to elevated levels of bradykinin, which is generated by the activation of the plasma contact system. Bradykinin binds to its B2receptor present on vascular endothelial cells, which leads to increased vascular permeability and subsequent tissue edema^[4,5]^.

HAE is mainly divided into three categories:

- HAE Type I: This type accounts for approximately 85% of the patients and occurs due to missense, nonsense, deletion, or insertion mutations in the SERPING1 gene on chromosome 11. This results in the formation of misfolded or truncated C1-INH protein. Clinical features include edema, respiratory distress, and abdominal pain^[4]^.
- HAE Type II: Type II HAE is usually associated with missense mutations in the SERPING1 gene. This variety, on the other hand, has normal or high C1-INH levels, but C1-INH is functionally defective^[4]^.
- HAE Type III: Type III HAE, also known as HAE with normal C1-INH, usually occurs in women and is linked to abnormalities in the F12 gene, which codes for coagulation factor XII. Despite normal C1-INH levels and function, these mutations increase factor XII activity, which leads to increased bradykinin production. Hormonal variables like estrogen may have an impact on the illness, and some people with this type may not have detectable genetic abnormalities^[7]^.

The mutational spectrum of HAE is still being expanded by recent genetic research. For instance, several afflicted members of a single pedigree were found to have c.708T>G (p.Phe236Leu), a new heterozygous SERPING1 mutation that confirmed autosomal dominant inheritance and showed co-segregation of genotype and phenotype. These results demonstrate how particular variations might enhance comprehension of clinical variability and increase diagnostic accuracy^[8]^.

At the population level, a 2023 Latvian nationwide survey further confirmed this heterogeneity, reporting diverse SERPING1 variants and frequent diagnostic delays (Kanepa *et al*^[9]^). This highlights the importance of incorporating genetic results into patient management and diagnosis.

Importantly, both family- and population-based studies underscore that biochemical parameters alone are not always consistent. For example, even among individuals with the same mutation (c.708T>G, p.Phe236Leu), C1-INH and C4 levels varied below, within, or above the normal range (Jiang *et al*^[8]^). This emphasizes how variables like medication history, disease status, and sample schedule affect lab results. As a result, genetic testing is increasingly acknowledged as being crucial for verifying HAE and preventing misdiagnosis, even though biochemical tests are still necessary^[8]^.

## Current management and treatment options for HAE

The main target of medical intervention is to prevent life-threatening complications of HAE. Involvement of organs such as the larynx and GI can pose a significant risk of asphyxia due to laryngeal edema and severe pain secondary to gastric involvement^[4]^.

### Acute management

Acute AE attacks usually resolve on their own within 3–5 days. However, complications such as laryngeal edema, asphyxiation, and decapitating pain secondary to gastrointestinal attacks are of concern. In cases of these symptoms, medical intervention is targeted solely to reduce the risk of complications and severity, if initiated within 6 hours of symptom onset. Long-term therapy is directed toward identifying precipitating factors and improving the patient’s quality of life^[4]^.

#### C1-INH administration

Recombinant human C1-INH and plasma-driven C1-INH are the two forms of C1-INH that can be administered in an acute setting. After subcutaneous administration, symptoms subside within an hour and are relieved within 2 h^[4]^. Upon their unavailability, FFP with C1-INH can be used as an alternative; however, considering its efficacy and side effects profile, it is usually not recommended. The IV formulations of these two agents are preferred in a hospital setting under the brand names Cinryze or Berinert. The side effect mostly associated with its use is headache. RhC1-INH, derived from the milk of transgenic rabbits, has low efficacy and thus it demands larger doses than pdC1-INH^[5]^.

### Bradykinin receptor antagonist

Icatibant, a B2 receptor antagonist of bradykinin, is also used; the dosing of which is weight-based, routinely administered as a 30 mg SC injection in patients aged 65 or above^[5]^. However, this drug should be used cautiously due to the reported decrease in cardiac blood flow^[10]^.

#### Recombinant plasma kallikrein inhibitors

Ecallantide, a subcutaneous agent used in patients aged 12 or above, is usually administered in a hospital setting due to drug reactions and anaphylaxis reports. The drug works by blocking the production of bradykinin.

Taking into account the clinical efficacy and safety profile of acute treatment options, pdC1-INH (IV) is the agent of choice, while RhC1-INH (IV) is similar in efficacy; however, it is a shorter-acting agent. Subcutaneous agents are also effective second in line; FFP (IV) is reserved as a backup because of its slower action. Since angioedema attacks are precipitated by elevated bradykinin levels, treatments such as corticosteroids, antihistamines, and epinephrine are ineffective^[4,5]^. Similarly, ACE inhibitors should be discontinued due to their role in increasing bradykinin levels, which can exacerbate angioedema. Symptomatic management also involves the use of anti-diarrheal agents, antiemetics, or laxatives for gastrointestinal symptoms^[5]^.

#### Prophylactic management

Prophylaxis in HAE is directed especially in stressful and traumatic situations, such as before major dental work or endoscopic surgeries and intubations. A plasma-derived C1-INH formulation (pdC1-INH), Haegarda, can be used prophylactically. Tranexamic acid (TXA), an antifibrinolytic agent, is used for both acute management and short-term prophylaxis^[4]^. The population selection for long-term prophylaxis is based on confounding factors such as severity, frequency of attacks, treatment availability, and comorbidities^[5]^.

#### Plasma kallikrein inhibitors

Monoclonal plasma kallikrein inhibitor named Lanadelumab, and pdC1-INH agents like Cinryze and Haegarda are recommended first-line agents^[4]^. Oral plasma kallikrein inhibitor, namely, Berotralstat, is approved for use in patients aged 12 or above; however, it comes with an increased risk of QT prolongation^[11]^.

#### Androgenic agents

Anabolic androgenic agents such as danazol and an antifibrinolytic agent like TXA are second-line agents and are also used prophylactically. However, the precise mechanism of its effect on AE is unknown, but it is known to increase C1-INH levels. Although these drugs are second-line agents, their use is still discouraged in prepubertal males and pregnant patients following their known side-effect profile^[12]^.

It is to be noted that these therapies have their own limitations, as they provide symptomatic relief in acute complicated states, and do not affect the actual genetic background of the disease. Apart from this, the need for repeated administration and variable individual response makes it inconvenient for the patients. For short-term prophylaxis, pdC1-INH (IV/SC) is often the best choice; androgens work but are limited by their side effects; TXA is less effective and is used if other options are unavailable. For long-term prophylaxis, the preferred options are lanadelumab, subcutaneous or intravenous plasma-derived C1-INH, while berotralstat offers an oral alternative but carries a risk of QT prolongation. Androgens and TXA are considered second-line choices due to safety limitations. These limitations provide a framework for future interventions such as gene editing therapies, which not only address these concerns but also provide a curable long-term solution to the underlying genetic cause of this disease.

## Future directions – CRISPR-based gene editing for HAE

**Figure 1.**
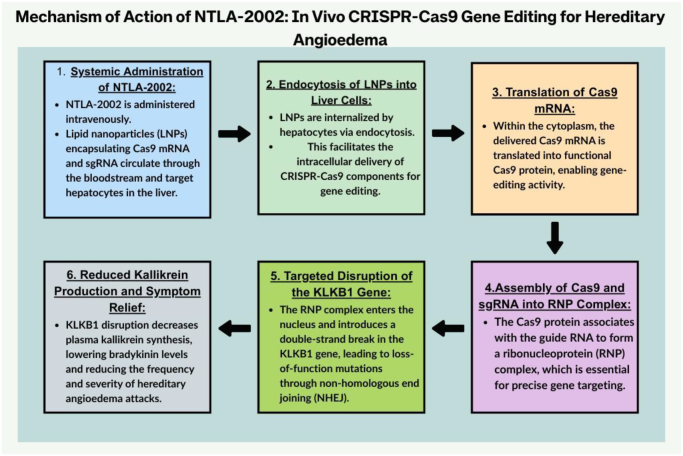
Mechanism of action of NTLA-2002: in vivo CRISPR-Cas9 gene editing for hereditary.

While current therapies focus on symptomatic control and prophylaxis, emerging approaches such as gene editing aim to address the underlying cause of HAE at the genetic level. NTLA-2002 is an *in vivo* gene-editing therapy based on clustered regularly interspaced short palindromic repeats (CRISPR)–Cas9. In this approach, CRISPR-Cas9 is delivered to the liver using lipid nanoparticles, where the *KLKB1* gene is mainly expressed^[3]^. Inside the liver cells, Cas9 and sgRNA complex induce a double-strand break in the *KLKB1* gene at the target site^[3]^. The repairs result in disrupted genes leading to reduced production of prekallikrein and a drop in overall plasma kallikrein levels^[1]^. A diagrammatic representation of this mechanism is shown in Figure 1.

As a one-time treatment administered systemically, it has the potential to convert lifelong prophylaxis into a one-time intervention^[1,3]^. In contrast, current therapies – including C1-INH replacement, kallikrein inhibitors, lanadelumab, berotralstat, and icatibant – require repeated IV/SC or daily dosing, remain non-curative, and are associated with breakthrough attacks, adherence challenges, and sustained lifetime costs^[4,12]^. A comparison between conventional treatment options and CRISPR/Cas9 gene therapy is shown in Table 1.

**Table 1:** Comparison of conventional therapies and CRISPR-based gene-editing therapy (NTLA-2002) for hereditary angioedema

Feature	Conventional therapies (C1-INH, lanadelumab, berotralstat, icatibant)	CRISPR-Cas9 therapy (NTLA-2002)
Mechanism	Replaces the missing/defective C1 esterase inhibitor protein, inhibiting the kallikrein/bradykinin pathway to reduce attacks^[4]^.	Reduces plasma kallikrein levels by CRISPR-Cas9–mediated editing of the *KLKB1* gene^[1]^.
Mode of administration	C1-INH, lanadelumab: IV or SC injections	Single IV infusion with lipid nanoparticle delivery of CRISPR-Cas9^[1]^.
	Berotralstat: oral	
	Icatibant: SC^[4]^.	
Dosing frequency	C1-INH: prophylaxis every 3–4 days; on-demand IV for acute attacks.	One-time administration^[1]^.
	Lanadelumab: every 2 weeks	
	Berotralstat: once daily	
	Icatibant: on demand at attack onset; repeat every 6 h if needed (max 3 doses/24 h)^[4]^.	
Efficacy	Reduce the attack frequency^[4]^.	Dose-dependent reductions in plasma kallikrein activity and approximately 90%–95% reduction in monthly attack rate^[1]^.
Safety profile	Injection/Infusion-site reactions are common but generally well tolerated^[4]^.	Generally well-tolerated^[1]^.
Long-term outcomes	Required repeated dosing; sustained life time costs^[4]^.	Durable suppression of kallikrein activity and sustained prevention of attacks after just one infusion^[1]^.

## Clinical evaluation of NTLA-2002

A total of three trials, which include two multicenter and one single-center, open-label, clinical trial, have been conducted. A total of 63 patients were part of these trials, in which they were administered NTLA-2002 at a single dose of 25, 50, or 75 mg^[1,13]^. After a follow-up of 16 weeks, the drug was found to significantly reduce the attacks of angioedema along with alleviating its symptoms^[1]^. According to Hilary *et al*^[1]^, there was a significant reduction in the mean percentage changes of total plasma kallikrein protein level by 67% in the patients who were administered 25 mg, 84% in the patients receiving 50 mg, and 95% in the patients receiving 75 mg of NTLA-2002^[1]^. Similarly, an overall reduction of 95% in the mean percentage change for the frequency of angioedema attacks per month was reported^[1]^. Danny *et al*^[12]^ found a significant decrease in the estimated mean monthly attack rate of angioedema with 0.70 (95% confidence interval: 0.25–1.98) when 25 mg and 0.65 (95% confidence interval: 0.24–1.76) when 50 mg NTLA-2002 was given to the patients. The placebo group showed a significantly higher estimated mean monthly angioedema attack rate of 2.82 (95% confidence interval: 0.80–9.89). Also, the reduction in the mean percent change for the total plasma kallikrein protein levels was 55% among the 25 mg group and 86% among the 50 mg group^[1,13]^. In a single-center open-label first-ever human clinical trial, Longhurst *et al*^[1]^ also reported a significant reduction in mean attacks from week 5 to week 16 by 89% in the 25 mg group patients. Details are mentioned in Table 2.

**Table 2:** Results of the clinical trials

Name of study	Study design	Study population	Drug used	Follow-up for clinical efficacy	Efficacy	Safety	Adverse effect
Hilary *et al*^[1]^	Multicenter, open-label clinical trial (phase 1)	30 patients	NTLA^a^-2002 (25-, 50-, and 75-mg)	16 weeks	Significant reductions in mean percentage changes of total plasma kallikrein protein level: 67% in the 25-mg group, 84% in 50-mg group, 95% in 75-mg group. Significant reduction in mean percentage change for the number of angioedema attacks per month: 95% in the overall group, 91% in the 25-mg group, 97% in the 50-mg group, 80% in the 75-mg group.	No dose-limiting toxic effects were reported. Grade 1 Infusion-related reaction (in 70% patients) and fatigue (in 60% patients). One grade 2 infusion-related reaction. No grade 3 or higher adverse events.	Infusion-related reaction, fatigue, and back pain.
Danny *et al*^[12]^	Multicenter, open-label clinical trial (phase 2)	27 patients	NTLA-2002 (25-mg, 50-mg and placebo with 2:2:1 ratio)	16 weeks	Significant reduction in estimated mean monthly attack rate: 0.70 (0.25–1.98) for 25-mg 0.65 (0.24–1.76) for 50-mg versus 2.82 (0.80–9.89) for placebo Significant reductions in mean percentage changes of total plasma kallikrein protein level 55% in the 25-mg group 86% in 50-mg group.	N/A	Headache, fatigue, and nasopharyngitis
Longhurst *et al*^[13]^	Multicenter, open-label clinical trial (phase 1)	6 patients	NTLA-2002	16 weeks	Significant reduction in mean attacks from week 5 to week 16 of 89% in the 25-mg group	No severe adverse event reported.	The most frequent adverse events were mild infusion-related reactions and fatigue.

^a^ NTLA-2002 targets the gene encoding kallikrein B1.

Preclinical studies in animal models demonstrated that NTLA-2002 effectively disrupted the *KLKB1* gene in hepatocytes, resulting in durable suppression of plasma kallikrein levels without evidence of hepatotoxicity or immune-mediated adverse effects^[1]^. In early-phase human trials, single-dose NTLA-2002 produced a dose-dependent reduction in plasma kallikrein (67% reduction at 25 mg, 84% at 50 mg, and 95% at 75 mg), accompanied by a 95% overall reduction in the mean frequency of HAE attacks^[1,13]^. Across these studies, most participants remained attack-free during follow-up, and the majority of adverse events were mild, including transient infusion-related reactions and fatigue^[1,13]^. These findings collectively provide strong preliminary evidence supporting both the efficacy and safety of NTLA-2002.

It should be noted that the primary studies did not report *P*-values or statistical testing between dose groups; therefore, this review summarizes efficacy outcomes as presented in the original publications without additional statistical comparisons.

## Safety and tolerability

Across all clinical trials, the incidence of adverse systemic or dose-limiting toxicities was minimal. Only 7 out of 30 patients reported infusion-related reactions, which were mild with no further sequel. One patient reported a grade 2 infusion-related reaction. No grade 3 or higher adverse event was reported^[1]^. These findings strongly suggest that gene editing therapy is safe and well-tolerated within the context of reported clinical studies. However, as these studies were conducted in controlled environments, the actual rate of adverse outcomes in real-world practice may differ. Across all clinical trials, infusion-related reactions were the most common adverse event. Specifically, 7 out of 30 patients (23%) in the total trial population experienced infusion-related reactions, and among those who reported adverse events, infusion-related reactions accounted for approximately 70%. These reactions were generally mild, with only one patient experiencing a grade 2 reaction.

The most common adverse event was an infusion-related reaction seen in 70% of the patients, followed by fatigue seen in 60% of the patients. Also, back pain was commonly reported in these patients^[1]^. The second clinical trial also reported fatigue to be a common side effect, along with headaches and nasopharyngitis, in the patient population^[13]^. According to Longhurst *et al*^[1]^, the most frequent adverse events were mild infusion-related reactions and fatigue in their single-center clinical trial. Details are mentioned in Table 2. While NTLA-2002 appears to have a good safety profile, there is limited information on its pharmacokinetics, as it has not been extensively studied in humans. *In vitro* studies indicate the distribution of these lipid nanoparticles of NTLA-2002 to the liver, ensuring gene editing within hepatocytes. Following administration, the components of NTLA-2002 are designed to be rapidly cleared from the bloodstream, minimizing systemic exposure^[1,13]^. Further research into its pharmacokinetics is needed to ensure its optimal use. A detailed breakdown of adverse events by severity (Grades 1–4) was not reported in the primary studies; therefore, only limited safety data are available and have been summarized here as presented in the original publications.

Although NTLA-2002 demonstrated a favorable short-term safety profile, the long-term risks remain uncertain. Potential concerns include immune responses to Cas9 protein, hepatotoxicity related to lipid nanoparticle delivery, and unintended off-target gene edits, none of which were systematically assessed in early-phase trials. Ongoing and future studies with extended follow-up are needed to address these safety considerations.

## Discussion

As mentioned above, the recent introduction of NTLA-2002, a CRISPR-Cas9-based gene-editing therapy, presents a paradigm shift in the treatment of a potentially life-threatening disease named HAE^[1]^. Notably, CRISPR-Cas9 gene-editing has emerged as a potential one-time therapeutic approach. It directly targets the *KLKB1* gene in hepatocytes. Investigational therapies such as NTLA-2002 aim to durably reduce plasma prekallikrein and total plasma kallikrein levels in contrast to conventional therapies that require continuous dosing and provide only transient relief. Its mechanism involves targeted disruption of the *KLKB1* gene in hepatocytes, leading to decreased production of plasma kallikrein and bradykinin and thereby reducing angioedema attacks^[1,13]^.

A key consideration when evaluating NTLA-2002 is how the benefits of a one-time gene-editing therapy compare with existing long-term management strategies. Current treatments, such as C1-INH replacement, lanadelumab, and berotralstat, are effective in controlling HAE attacks but require frequent, lifelong administration and primarily address only symptoms rather than the underlying genetic defect^[4,5,11]^. In contrast, NTLA-2002 has demonstrated the potential to provide durable kallikrein suppression and major reductions in attack frequency after a single infusion, raising the prospect of longer-term disease modification or even functional cure^[1,13,14]^. This approach could significantly lower the treatment burden on patients and families, improve quality of life, and effectively lower cumulative healthcare costs. However, uncertainties regarding long-term durability, safety, and the high upfront cost of therapy remain important barriers^[14–16]^. Until these issues are resolved, conventional therapies will continue to serve as essential options, particularly as bridging or fallback treatments for patients not eligible for gene-editing interventions. Recent multicenter and single-center trials have demonstrated promising outcomes, including a 9% mean reduction in monthly HAE attack rates, with up to 73% of patients remaining attack-free at 16 weeks^[1,13]^. Extended follow-up data from interim analyses suggest durability of effect, with some patients maintaining attack-free intervals for nearly 10 months^[14]^. Importantly, the overall safety profile has been favorable, with most adverse events being mild and transient^[1,13]^. Taken together, these early findings suggest that CRISPR-based approaches could reduce disease burden and decrease reliance on repeated C1-INH or kallikrein inhibitor administration.

However, the possibility of off-target mutations remains a key theoretical concern. These may occur when guide RNAs bind partially homologous genomic regions, potentially leading to harmful oncogenic consequences^[16]^. Modern advances in high-fidelity Cas9 design and focused sgRNA specificity lower but do not completely eliminate this risk^[1,4,13]^. Currently available safety data are based on relatively short follow-up, and the long-term risks of *in vivo* gene editing remain uncertain. Potential delayed consequences include unintended genetic alterations, genomic instability, and immune responses, which may not emerge until years after treatment^[15]^. To address these concerns, future trials should incorporate extended monitoring, including genomic and transcriptomic analyses, to identify off-target edits and other persistent effects^[15,17]^.

Beyond biological risks, ethical considerations must also be taken into account. Concerns regarding inadvertent germline editing, patient consent, equitable access, and the societal consequences of gene-editing technologies remain unresolved^[1,15]^. While NTLA-2002 targets somatic cells only, regulatory frameworks must ensure rigorous oversight to safeguard against misuse and exploitation.

Cost burden and easy accessibility also remain significant challenges. NTLA-2002 is projected to be priced in line with other gene therapies (upward of USD 2.2 million per treatment), creating substantial challenges for patients and health systems, especially in resource-limited settings^[16,18]^. Although a one-time treatment may ultimately prove cost-saving compared to lifelong prophylaxis, the high upfront cost and complex manufacturing requirements hinder widespread adoption^[16,18]^.

### Limitations

While this review highlights the promising potential of NTLA-2002, several limitations should be considered. Available clinical data are restricted to early-phase trials with small sample sizes and short follow-up, limiting conclusions on long-term efficacy and safety. This review also included only studies published in English, which may introduce language bias. Moreover, as a narrative review, formal risk-of-bias or quality assessments were not performed, and the conclusions are dependent on the strength of the available data. Finally, given the rapidly evolving nature of CRISPR research, newer data may not have been captured at the time of writing.

## Conclusion

HAE is a rare genetic disorder with life-threatening complications. Current treatments largely provide symptomatic relief, whereas CRISPR-Cas9–based therapies such as NTLA-2002 offer the possibility of a one-time, disease-modifying approach. Early clinical trials have shown up to a 95% reduction in mean monthly attack frequency, with most patients remaining attack-free during follow-up. Despite these encouraging results, uncertainties remain regarding long-term safety, durability, and access. Future studies should address extended safety monitoring, broader patient populations, and real-world effectiveness to establish NTLA-2002’s role in practice.

## Data Availability

Not applicable.
